# Early Adherence to Antiretroviral Medication as a Predictor of Long-Term HIV Virological Suppression: Five-Year Follow Up of an Observational Cohort

**DOI:** 10.1371/journal.pone.0010460

**Published:** 2010-05-05

**Authors:** Nathan Ford, Marta Darder, Tim Spelman, Emi Maclean, Edward Mills, Andrew Boulle

**Affiliations:** 1 Médecins Sans Frontières, Cape Town, South Africa; 2 Burnet Institute, Melbourne, Australia; 3 Faculty of Health Sciences, University of Ottawa, Ottawa, Canada; 4 Centre for Infectious Diseases and Epidemiology Research, University of Cape Town, Cape Town, South Africa; University of California San Francisco, United States of America

## Abstract

**Objective:**

Previous studies have demonstrated a cross-sectional relationship between antiretroviral adherence and HIV virological suppression. We assessed the predictive value of baseline adherence in determining long-term virological failure.

**Design:**

We assessed baseline adherence via an adherence questionnaire between administered to all consenting patients attending antiretroviral clinics in Khayelitsha township, South Africa, between May 2002 and March 2004. Virological status was ascertained after five years of follow up and multivariate analysis used to model associations of baseline variables and medication adherence with time to viral suppression or failure.

**Results:**

Our adherence cohort comprised 207 patients, among whom 72% were female. Median age was 30 years and median CD4 count at initiation was 55 cells/mm^3^. We found no statistically significant differences between baseline characteristics and early adherence groups. Multivariate analysis adjusting for baseline CD4 and age found that patients with suboptimal baseline adherence had a hazard ratio of 2.82 (95% CI 1.19–6.66, p = 0.018) for progression to virological failure compared to those whose baseline adherence was considered optimal.

**Conclusions:**

Our longitudinal study provides further confirmation of adherence as a primary determinant of subsequent confirmed virological failure, and serves as a reminder of the importance of initial early investments in adherence counseling and support as an effective way to maximize long-term treatment success.

## Introduction

The widespread availability of antiretroviral therapy (ART) has changed the course of HIV infection in developed countries, and comparable benefits are observed in resource-limited settings. The provision of effective ART is increasingly understood to be critical for both medical and a public health reasons. Maintaining virological suppression is an important objective for both the individual (reduced morbidity and mortality) and at the population level (reduced resistance [1] and transmission [2]).

A mixture of biologic factors such as virus type, host immunology, disease status and genetics, together with characteristics of medications such as drug potency, toxicity, formulation, and pharmacology can influence adherence and therapeutic success. Thus, virological failure may result from suboptimal adherence, poor drug potency, drug resistance, or a combination of these factors [3].

Amid these multiple explanations, sub-optimal adherence to medication has been recognized as one of the main patient-mediated risk factors for treatment failure [3] and several studies have demonstrated a cross-sectional relationship between adherence and virological suppression [4]–[7]. It is unknown whether patient-mediated factors may predict poor adherence, and thus poor virological suppression, in the long-term. We aimed to assess this relationship in a longtitudinal study to determine the predictive value of baseline adherence in determining virological failure over time.

## Methods

### Study Setting and data sources

Our study includes patients enrolled in an HIV treatment programme, in Khayelitsha township, South Africa. ART was first provided through a pilot demonstration project in May 2001, with initial capacity to provide ART for 180 adults. By the end of 2007, the service had cumulatively enrolled over 7000 adults onto ART as part of the routine programme [8].

We used data derived from a baseline adherence questionnaire done in Khayelitshsa township during the early phase of antiretroviral provision in 2002. This adherence study was conducted at a time when the ability of people in Africa to adhere to antiretroviral medication was questioned, a hypothesis that has since been found to be unsupported by evidence [9].

The adherence survey included all consenting patients enrolled onto antiretroviral therapy at primary care clinics in Khayelitshsa township, South Africa between May 2002 and March 2004. Self-reported adherence was assessed by a dedicated study team unrelated to the provision of clinical care using a modified version of the AIDS Clinical Trials Group questionnaire [10] that was forward- and back-translated and piloted prior to administration. We assessed adherence one and three months after initiation of ART and considered patients as highly adherent if they reported ≥95% adherence to medication; otherwise, adherence was considered as suboptimal.

Baseline and outcome data were collected as standard indicators for monitoring and evaluation in the Khayelitsha programme. Viral load (NucliSens EasyQ HIV-1 assay (bioMerieux, Boxtel, The Netherlands) and CD4 count (single-platform panleucogating method) were assessed at baseline and every six months according to manufacturer's instructions. Virological failure was defined as two consecutive HIV RNA levels greater than 5000 copies/ml, in accordance with national guidelines. Mortality ascertainment is corrected through linkages with the South African vital registration system [8]. The duration of follow-up for this study was five years.

### Statistical analyses

Descriptive analyses were based on percentages and frequencies for categorical variables and medians and interquartile ranges (IQR) for continuous variables. Continuous variables were assessed for skew and as all demonstrated non-normality they were compared using the Wilcoxon rank-sum test. Proportions were compared using the χ^2^test and, in the case of small numbers, the Fisher's Exact test. We used Nelson-Aalen cumulative hazards estimates to describe time to confirmed virological failure per adherence group, as this method provides a appropriate summary for failure events [11]. Univariate cox regression was used to model the individual associations of baseline variables and medication adherence with time to viral suppression or failure; variables were stratified into discrete categories as follows: early adherence (<95% or ≥95%), baseline CD4 (<50 cells/mm^3^ or ≥50 cells/mm^3^), sex (male or female), HAART regimen (efavirenz- or nevirapine-based), and age (per 10 years). Multivariate models of associations with virological failure included variables associated with poor adherence in univariate analysis adjusted for potential confounders identified a priori. Hazard proportionality was assessed by analysis of scaled Schoenfeld residuals. All reported p values are exact and 2-tailed, and for each analysis p<0.05 was considered significant. All analyses were performed using STATA version 11.0 (StataCorp, College Station, Texas).

All aspects of data collection (adherence questionnaire, analysis of routine cohort data and the linkage to the national death registry) were approved by the University of Cape Town Research Ethics Committee. As the data is based out routinely collected data and anonymized, informed consent was not sought.

## Results

Our adherence cohort comprised 207 patients, among whom 149 (72%) were female. The median age at ART initiation was 30 years (IQR 28–37) years and the majority (80%) received an efavirenz-based regimen. Median CD4 count at initiation was 55 cells/mm^3^ (IQR 20–115 cells/mm^3^) and median HIV-1 RNA levels at initiation was 5.03 log_10_ copies/mL (IQR 4.3–5.5 log_10_ copies/mL). Our early adherence assessment found that 181 (87%) patients were considered highly adherent. We found no statistically significant differences between baseline characteristics and early adherence groups (Table 1).

**Table 1:** Patient characteristics.

Category	Subcategory	Total patients (n = 207)	Patients with adherence ≥95% (n = 181)	Patients with adherence <95% (n = 26)	p
Sex, n (%)	Male	58 (28)	50 (28)	8 (31)	0.74
	Female	149 (72)	131 (72)	18 (69)	
Age, median (IQR)		30 (28–37)	31 (8–37)	30 (26–32)	0.147
Baseline CD4, median (IQR)		55 (20–115)	51 (19–121)	59.5(23–102)	0.71
Baseline VL, median (IQR)		110,000 (21,000–310,000	110,000 (20,000–320-000)	155,550 (97,000–300,000)	0.19
Regimen, n (%)	EFV	165 (80)	144 (79)	21 (81)	0.24*
	NVP	40 (19)	36 (20)	4 (15)	
	Other	1 (1)	1 (1)	1 (4)	
Prior TB	Yes	98 (47)	87 (48)	11 (42)	0.58
	No	109 (53)	94 (52)	15 (58)	

*Fishers exact; EFV, efavirenz; NVP, nevirapine; TB, tuberculosis; VL, viral load; IQR, interquartile range.

**Figure 1:**
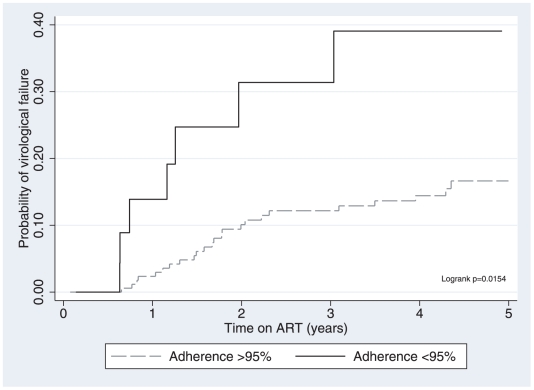
Cumulative hazard estimate for virological failure.

In our univariate analysis suboptimal early adherence was the only association with virological failure (hazard ratio 2.72, 95%CI 1.16–6.31, p = 0.02) (Table 2). In multivariate analysis we adjusted for baseline CD4 and age as these have been found to be associated with virological failure in larger studies from the same population [8]; this analysis found that patients with suboptimal baseline adherence had a hazard ratio of 2.82 for progression to virological failure compared to those whose baseline adherence was considered optimal (95% CI 1.19–6.66, p = 0.018). Cumulative hazard estimates by adherence category are described in Figure 1.

**Table 2:** Associations between baseline characteristics and time to virological failure.

Predictor variable	Category	Failures n, %	Univariate		Multivariate	
			HR (95%CI)	p	HR (95%CI)	p
Adherence	>95%	25 (78.1%)	1		1	
	<95%	7 (21.9%)	2.72 (1.17–6.31)	0.020	2.82 (1.19–6.66)	0.018
Sex	Male	12 (27.5%)	1			
	Female	20 (62.5%)	0.98 (0.93–1.03)*	0.12		
Age	20–29	15 (46.9%)	0.98 (0.93–1.03)*	0.35	0.98 (0.93–1.02)	0.43
	30–39	13 (40.6%)				
	40–49	2 (6.3%)				
	50–59	2 (6.3%)				
Baseline CD4	≤0.5×10^9^ cells/L	17 (53.1%)	1		1	
	>0.5×10^9^ cells/L	15 (46.9%)	0.79 (0.39–1.60)	0.52	0.69 (0.34–1.43)	0.32
Regimen	NVP	6 (18.7%)	1			
	EFV	26 (81.3%)	1.08 (0.44–2.94)	0.87		

*per category; EFV, efavirenz; NVP, nevirapine.

schoenfelds p = 0.12.

## Discussion

Our longitudinal study provides further confirmation of adherence as a primary determinant of subsequent confirmed virological failure, reinforcing the findings of previous studies that associate adherence with viraemia at a single point in time concurrent to or soon after the adherence measures [12].

We found that early adherence was a more important predictor of long-term virological suppression than prognostic variables such as CD4 and drug regimens, a phenomenon also observed in better-resourced settings [13]. Patients initiating ART and surviving the first three months of therapy typically have improved survival outcomes in the long term if they can maintain optimal adherence [14].

The use of routine viral load monitoring in South Africa enables the exploration of associations with confirmed virological failure. The confirmation of failure, subsequent to a period of adherence optimization, has been shown to identify patients with a high probability of multi-class drug resistance [15].

Our study was not able to distinguish whether the association between virological failure and early adherence was due to the early adherence being a marker of long term sup-optimal adherence, or due to ongoing viral replication under selective drug pressure in the early months on ART. Nevertheless, the association we describe validates the structured adherence interventions that are commonplace in the public health approach to ART, including facility-based counseling [16], and the use of early viral load testing to identify patients who might benefit from further adherence-promoting interventions [17].

Several issues need to be considered with respect to the external validity of our findings. First, there is no agreed definition of what constitutes sub-optimal adherence. We applied a conservative cut-off of 95% partly because the drug regimes used did not include boosted protease inhibitors, and partly because of the expectation of poor adherence. Nevertheless, the fact that the majority of our cohort (87%) was considered to be highly adherent is consistent with other findings that display, on average, good adherence within large patient populations [18]. Second, the use of self-reports in assessing medication adherence is subject to information (recall) bias. There is no gold standard for medication adherence, but studies from similar settings in southern Africa have found that self-report can provide a reliable measure compared to other methods [19], especially when administered by independent researchers rather than members of the clinical team. Finally, our adherence cohort was established during the early phase of the programme when most patients were severely immuno-compromized at the start of ART, as indicated by low median baseline CD4. In general baseline CD4 at ART initiation is today much higher than in previous years, both in this cohort [8] and other cohorts [20]. Low baseline CD4 has been found to be associated with virological failure [21], although the extent to which this relates to poor adherence is not known. The patients we describe had to take treatment at least twice daily, at least two different tablets, and often with uneven dosing. They were at relatively high risk of haematological and hypersensitivity reactions [22]. While great strides have been made in improving access to treatments in South Africa, we remain far from providing optimal treatments for patients from an adherence perspective.

As ART programmes mature and lessons emerge over time, concern is growing around the challenges of sustaining long-term adherence [23]. Our study serves as a reminder that a patients' initial experience with antiretroviral medication is critically important. Thus while models of care need to be developed to support ART care over time, the initial early investments in adherence counseling and support is an effective way to maximize the likelihood of treatment success in the longer term.

## References

[1] Gill VS, Lima VD, Zhang W, Wynhoven B, Yip B (2010). Improved virological outcomes in British Columbia concomitant with decreasing incidence of HIV type 1 drug resistance detection.. Clin Infect Dis.

[2] Granich R, Gilks C, Dye C, De Cock K, Williams B (2009). Universal voluntary HIV testing with immediate antiretroviral therapy as a strategy for elimination of HIV transmission: a mathematical model.. Lancet.

[3] Friedland GH (2006). HIV medication adherence. The intersection of biomedical, behavioral, and social science research and clinical practice.. J Acquir Immune Defic Syndr.

[4] Arnsten JH, Demas PA, Farzadegan H, Grant RW, Gourevitch MN (2001). Antiretroviral therapy adherence and viral suppression in HIV-infected drug users: comparison of self-report and electronic monitoring.. Clin Infect Dis.

[5] Nachega JB, Hislop M, Dowdy DW, Chaisson RE, Regensberg L (2007). Adherence to nonnucleoside reverse transcriptase inhibitor-based HIV therapy and virologic outcomes.. Ann Intern Med.

[6] Martin M, Del Cacho E, Codina C, Tuset M, De Lazzari E (2008). Relationship between adherence level, type of the antiretroviral regimen, and plasma HIV type 1 RNA viral load: a prospective cohort study.. AIDS Res Hum Retroviruses.

[7] Garcia R, Badaró R, Netto EM, Silva M, Amorin FS (2006). Cross-sectional study to evaluate factors associated with adherence to antiretroviral therapy by Brazilian HIV-infected patients.. AIDS Res Hum Retroviruses.

[8] Boulle A, Van Cutsem G, Hilderbrand K, Cragg C, Abrahams M (2010). Seven-year experience of a primary care antiretroviral treatment programme in Khayelitsha, South Africa.. AIDS.

[9] Mills EJ, Nachega JB, Buchan I, Orbinski J, Attaran A (2006). Adherence to antiretroviral therapy in sub-Saharan Africa and North America: a meta-analysis.. JAMA.

[10] Chesney MA, Ickovics JR, Chambers DB, Gifford AL, Neidig J (2000). Self-reported adherence to antiretroviral medications among participants in HIV clinic trials: the AACTG adherence instruments.. AIDS Care.

[11] Colosimo E, Ferreira F, Oliveira M, Sousa C (2002). Empirical comparisons between Kaplan-Meier and Nelson-Aalen survival function estimators.. J Statistical Computation Simulation.

[12] Ahoua L, Guenther G, Pinoges L, Anguzu P, Chaix ML (2009). Risk factors for virological failure and subtherapeutic antiretroviral drug concentrations in HIV-positive adults treated in rural northwestern Uganda.. BMC Infect Dis.

[13] Wood E, Hogg RS, Yip B, Harrigan PR, O'Shaughnessy MV (2003). Effect of medication adherence on survival of HIV-infected adults who start highly active antiretroviral therapy when the CD4+ cell count is 0.200 to 0.350×10(9) cells/L.. Ann Intern Med.

[14] Braitstein M, Brinkhof F, Dabis F, Schechter M, Boulle A (2006). Mortality of HIV-1-infected patients in the first year of antiretroviral therapy: comparison between low-income and high-income countries.. Lancet.

[15] Orrell C, Walensky RP, Losina E, Pitt J, Freedberg KA (2009). HIV type-1 clade C resistance genotypes in treatment-naive patients and after first virological failure in a large community antiretroviral therapy programme.. Antivir Ther.

[16] Coetzee D, Hildebrand K, Boulle A, Maartens G, Louis F (2004). Outcomes after two years of providing antiretroviral treatment in Khayelitsha, South Africa.. AIDS.

[17] Calmy A, Ford N, Hirschel B, Reynolds SJ, Lynen L (2007). HIV viral load monitoring in resource-limited regions: optional or necessary?. Clin Inf Dis.

[18] Ford N, Nachega J, Engel M, Mills E (2009). Directly observed antiretroviral therapy: a systematic review and meta-analysis of randomized clinical trials.. Lancet.

[19] Oyugi JH, Byakika-Tusiime J, Charlebois ED, Kityo C, Mugerwa R (2004). Multiple validated measures of adherence indicate high levels of adherence to generic HIV antiretroviral therapy in a resource-limited setting.. J Acquir Immune Defic Syndr.

[20] The ART-LINC Collaboration of the International Databases to Evaluate AIDS (IeDEA) (2008). Antiretroviral therapy in resource-limited settings 1996 to 2006: patient characteristics, treatment regimens and monitoring in sub-Saharan Africa, Asia and Latin America.. Trop Med Int Health.

[21] Boulle A, van Cutsem G, Hilderbrand K, Cragg C, Abrahams M (2009). Good outcomes on ART sustained at five years in Khayelitsha in spite of massive scale up..

[22] Boulle A, Orrel C, Kaplan R, Van Cutsem G, McNally M (2007). Substitutions due to antiretroviral toxicity or contraindication in the first 3 years of antiretroviral therapy in a large South African cohort.. Antivir Ther.

[23] Byakika-Tusiime J, Crane J, Oyugi JH, Ragland K, Kawuma A (2009). Longitudinal antiretroviral adherence in HIVþ Ugandan parents and their children initiating HAART in the MTCT-Plus family treatment model: role of depression in declining adherence over time.. AIDS Behav.

